# Old age satisfaction regarding geriatric home services in Erbil city

**Published:** 2015

**Authors:** MA Sangar, FA Karem, NN Alireza, AK Muaf

**Affiliations:** * Hawler Medical University, Nursing College, Erbil, Kurdistan Region/ Iraq,; ** Department of Medical Surgical Nursing, School of Nursing and Midwifery, Tehran University of Medical Sciences, International Campus (TUMS-IC), Tehran, Iran

**Keywords:** old age, life satisfaction, geriatric home services, Erbil

## Abstract

**Background and objectives:** Life satisfaction is a vital imaginary situation in the psychosocial investigation of aging. Life pleasure is a multi-rule issue that relies on many objective and subjective components. In this research, the achievements are based on socio-demographic characteristics and old age satisfaction toward services in Geriatric home.

**Methods:** It is a cross-sectional study which had been conducted in Geriatric home service in Erbil city during the period from 27/6/2014 to 4/2/2015. A questionnaire was made including sections for demographic characteristics, satisfaction with living conditions utilizing a face-to-face interview format. 25 males and females of old ages were interviewed. Each interview was used as the method of data collection.

**Results:** Twenty-five old aged persons participated in this research in the geriatric home center in Erbil governorate. The greater part of them were males; age group was 52-70 years and single. The average duration of staying was of 1-6 years (68%). Many of them did not own friends outside the geriatric home and could not get in touch with their family.

**Conclusion:** Interventions must be organized to raise living satisfaction between old persons. Appropriate old age plans including important answers to the difficulties of the aged people are important to make them feel the element of culture.

## Introduction

Living enjoyment continues to be an essential construct in the psychosocial research of aging. It is one of the subjective conditions of the status of life, which is the very commonly approved and appears to be one of the facets of victorious aging, both of them being key concepts in aging. 

The financial arrangement, the erosion of social values, abating of social values, and social organizations like the union family were changed based on the urbanization, modernization, and globalization. Research reports were about life satisfaction, which is greatly dependent on psychosocial and socio-demographicpsychosocial variables [**2**].

Changes in the human body might cause difficulty in life. Frequently, that is why persons get in long-period care societies. For some citizens, aging was very dangerous or dismal. Others adapted well. The style you work with people can make them think better about themselves—and can make your act more favorable [**5**].

The aging procedure is, of course, a natural situation, which has its own nature; largely, it is not under the human’s control. But, it is additionally subject to the arrangements by which each community makes sense about the old lifetime. In the advanced countries, the chronological duration works a supreme position. The age of 60 or 65, is approximately equal to retirement years in most advanced countries wherever it is supposed to be the start of old age [**3**].

Most of the advanced countries have believed that the chronologic lifetime of 65 years as a determination of "elderly" or older people, but similar to common westernized ideas, this does not conform well to the condition in Africa. While this determination is slightly arbitrary, it is common times connected with the lifetime at which a person can start to take payment benefits. At the time, there is no United Nations official numerical model, but the UN accepted stoppage is 60+ ages to mention to the elderly people [**4**,**6**].

Older people which are not capable of handling the everyday life individually may have an another way of satisfaction than others with maintained self-care capability. It might well be that the transformation from being normal and self-supporting of aid self-care capability changes the scene of features contributing to the actions of regular living to becoming to live with a reduced life satisfaction [**1**].

The new socio-economic arrangement creates a severe social difficulty for old people. Programmed and deliberate actions, that will helpfully involve aged people correspond to their ability, must be established. Those old people who are experiencing illness require specific helps in their old age houses [**7**,**8**]. 

There is no huge geriatric home service in each city in Kurdistan. There is just one public Geriatric home in Erbil. The purpose of the research was to recognize the socio-demographic features and elder person satisfaction toward services in the geriatric home in Erbil city.

## Methodology 

A cross-sectional research design was adapted to recognize the socio demographic characteristics and home care services satisfaction by older age. The duration was from September 1, 2014 to June 5, 2015. Half of the elder age people in the geriatric home center were excluded because many of the disabled were not capable of joining in the research. A total representation size of 25 was selected to use a face-to-face interview format. 

A questionnaire which covered demographic features of the older lifetime (gender, age, education, religion, having children, children, duration, number of friends in the same place, having a friend outside the geriatric home, in-touch with family member) was designed by the researchers and the second section of the questionnaire covered the satisfaction of the house care services. 

Permission was taken by the manager of the geriatric home center, then a verbal consent was taken from whole the old aged persons who were capable of engaging in the research before starting the interviews.

An interview was used as a method of information gathering from both old aged genders. Further, the research was accepted by the moral committee of the college of nursing in Erbil. Data were examined by employing the statistical package for social sciences (SPSS, version 19). 

## Results of the research 

**Table 1** shows the most of the sample, 56% (n=14), were between age group 52-70 years old. Regarding the sex, most of the members, 88% (n=22), were male, in items of marital status, most of the members, 48% (n=12), were single/ never married, the majority of them, 84% (n=19), did not own children. With consideration of the address, 52%( n=13), were from the city, regarding the duration of staying in the geriatric home center, 68% (n=17) were between 1-6 years. 52% (n=13) were the participants who never attended school. 

44% (n=11) of them, of 4-6 years old were living together in same room, 72% (n=18) did not have friends outside the home center and 64% (n=16) did not have any connection with their family. 

**Table 1:** Socio demographic features of the old lifetime

Sociodemographic data		n=25	
		F	%
Age	33-51	2	8.0
	52-70	14	56.0
	71-89	9	36.0
Sex	Male	22	88.0
	Female	3	12.0
Marital Status	Single	12	48.0
	Married	10	40.0
	Divorce	1	4.0
	Others	2	8.0
Level of Training	Illiterate	13	52.0
	Primary	12	48.0
Address	City	13	52.0
	Suburb	12	48.0
Religion	Muslim	23	92.0
	Christian	2	8.0
Having Children	No	19	76.0
	Yes	6	24.0
Children	No children	19	84.0
	1-3	1	4.0
	4-6	2	8.0
	7-10	3	4.0
Duration	<1Year	2	8.0
	1-6Year	17	68.0
	7-12Year	4	16.0
	13-18 Year	1	4.0
	>19Year	1	4.0
Number of friends in the same place	0-3	10	40.0
	4-6	11	44.0
	7-10	4	16.0
Having friend outside of geriatric home	No	18	72.0
	Yes	7	28.0
In-touch with family member	No	16	64.0
	Yes	9	36.0

**Table 2** shows that 64% of participants were satisfied, 32% were fairly satisfied and 1% of them were poorly satisfied with the health care aids in the home center. With consideration of the nutrition program stratification, 76% were satisfied, 20% were fairly satisfied and 4% were poorly satisfied. Most of them, 76%, were satisfied with the room services and 24% of the participants were fairly satisfied.

With regard to the geriatric home environment, 56% of them were fairly satisfied, 28% of the elderly people were poorly satisfied with the environment. Regarding the availability of having bathing services, 88% of them were satisfied, 12% of the participants were fairly satisfied with all the services and 84% of them were satisfied by the general hygiene services and 60% of the aged persons in the geriatric home center were satisfied with the clothing services.

Many of the aged people, 80%, had a good connection with the health staff and the administration services. 

At the same moment, 60% of the aged persons were fairly satisfied with the general social exercise and entertainment program in the geriatric home center. With consideration of the safety measure, 48% were fairly satisfied, 40% of them were poorly satisfied and 12% of the aged people were satisfied.

Regarding the stratification from the transportation for the geriatric home resident for daily needs, 56% were fairly satisfied, 36% of the participants were poorly satisfied and 8% of the aged persons were satisfied by the services.

**Table 2:** Geriatric home services. Satisfaction according to residents:

Items	N=25						MS
	Good		Fair		Poor		
	F	%	F	%	F	%	
Health Services	16	64.0	8	32.0	1	4.0	1.40
Nutrition Program	19	76.0	5	20.0	1	4.0	1.28
Room services	19	76.0	6	24.0	0	0.0	1.24
Geriatric home environment	4	16.0	14	56.0	7	28.0	2.12
Bathing	22	88.0	3	12.0	0	0.0	1.12
Hygiene	21	84.0	3	12.0	1	4.0	1.20
Clothing	15	60.0	8	32.0	2	8.0	1.48
Relation with Health workers	20	80.0	4	16.0	1	4.0	1.24
Relationship with administration services	20	80.0	5	20.0	0	0.0	1.20
Social activity/ Entertainment	3	12.0	15	60.0	7	28.0	2.16
Safety measure	3	12.0	12	48.0	10	40.0	2.28
Transportation	2	8.0	14	56.0	9	36.0	2.28

## Discussion

Traditionally, in Iraqi Kurdistan, as in many other Mediterranean countries, older people in need have often lived with other family members, but nowadays the situation is beginning to change. This includes migration, urbanization, war, changing culture and increasingly divergent values between young and old generations, and communal and commercial deprivation. 

Based on the policy of Geriatric Home Center in Erbil, the lifetime of elders who attend the home center for females should be 55 years and over, and males 65 and above but sometimes, there is some exclusion depending on the situation of the elder. 

The current study confirmed that most of them were between the group age 52-70 years, which represented, males, who never got married. This outcome matched with the research done in Jordan in which 29.3% of the elders in the nursing home were single [**8**].

Education has an important role in designing the future of the community. Regarding the elders’ education level, many of them, 52%, were illiterate. This outcome matched with the research which determined that the majority of elders, 25 (35.2%), did not receive any formal education. Illiteracy was 19.7% in women subjects, and 15.5 % in men [**9**].

From the city center and Muslim, most of them did not have children, nearly 84%, while most of them had friends in the same room and outside the home and most of them were in touch with family, and the longest duration of stay of old adults in the Geriatric home was between 1-6 years, 68%, so, the interpretation of these results is the following: the standard age of old males was 65 years and because most of them did not have children to follow them up, they came to that home, the study being in agreement with study of [**10**,**12**,**14**], who noticed that the aged men came to the geriatric home to see the services and since there was no one to supervise and follow them up in their living. 

Some results showed that most of them were satisfied with most health services like (Nutrition Program 76%, Room services 76%, Bathing services 88%, Hygiene 84%, Clothing 60%, and Relationship with the administration services 80%) so, these services were good. These outcomes were in accordance with the studies done by [**13**,**15**], who mentioned that services were more important to satisfy the aged male and females in the Geriatric home.

Other results showed that there were poor services in the geriatric home, such as Geriatric home environment, Social activity/ Entertainment, Safety measures, and Transportation services, so, these results were in accordance with the study done by [**11**,**16**,**17**], who mentioned that poor services, and the quality of food and socialization were an effect on satisfaction among residents in the Geriatric home.

Providing comfortable and clean bedroom, treating residents with respect, providing sufficient and suitable food, having an in-geriatric home specialist physician, an adequate number of nurses and having more recreational exercises in the home are important for all the elderly. These actions developed the need for thegeriatric homes to improve their quality of service. The engagement in daily activities is important for the residents in the geriatric homes. Other than the usual leisure activities, it is important to encourage the public to visit the geriatric home center. School, university / college students, and volunteer people can arrange for their members to visit the geriatric home and have some programs for the aged. Thus, the community will be more aware of the problems linked to the support and attention of the aged.

## Conclusion 

Interventions require being designed to increase life satisfaction among old persons. Appropriate old age management involving important answers to the difficulties of the aged persons are important to give them feel the element of culture. Many of the elders are pleasured with the Geriatric home services including (nutrition, room services, bathing, hygiene, clothing, and relationship with staff in the home) while they are not satisfied with some items of services such as environment, social activities, entertainment, safety measures and transportation.

**Recommendation**

Encouragement of the Ministry of Work and Social Affairs to improve services in the Geriatric home especially regarding the environment, social activities, entertainment, safety measures and transportation services as it was concluded in the outcomes of study.
